# Impact of acute caffeine intake on local tolerance to cold before and after total sleep deprivation

**DOI:** 10.1113/EP092356

**Published:** 2025-03-27

**Authors:** Baptiste de Lorgeril, Pierre‐Emmanuel Tardo‐Dino, Cyprien Bourrilhon, Michael Quiquempoix, Catherine Drogou, Lise Mateo, Mégane Erblang, Philippe Colin, Pascal Van Beers, Mounir Chennaoui, Danielle Gomez‐Merino, Fabien Sauvet

**Affiliations:** ^1^ Ecole du Val de Grâce Paris France; ^2^ Hôpital d'instruction des armées Laveran Marseille France; ^3^ UMR LBEPS Université d'Evry Paris France; ^4^ Institut de recherche biomédicale des armées (IRBA) Brétigny sur Orge France; ^5^ EA7330 VIFASOM Université de Paris Paris France; ^6^ Hôpital d'instruction des armés Legouest Brest France

**Keywords:** caffeine, cold‐water immersion, finger skin blood flow, finger skin temperature, laser speckle, pain, sleep deprivation

## Abstract

Total sleep deprivation (TSD) alters local cold tolerance and could thus increase the risk of cold injury. We evaluated the impact of acute caffeine intake, the main countermeasure to TSD‐related deleterious effects, on local cold tolerance before and after TSD. Thirty‐six healthy subjects underwent two TSD protocols (i.e., continuous wakefulness), with randomized crossover intake of acute caffeine or placebo (2.5 mg/kg) administered twice during wakefulness. Before and after 33 h of TSD, finger (index and annular) temperature and skin blood flow were assessed during cold‐water immersion (CWI, 5°C, 20 min) followed by 20 min of rewarming in ambient air. We showed no significant effects of TSD on mean finger temperature during CWI in the placebo condition, but a significant reduction of the minimal temperature (8.86°C ± 0.35°C vs. 8.64°C ± 0.27°C, *p* = 0.02). During rewarming, we showed a reduction in temperature in the placebo condition (*p* = 0.02 for the mean temperature and *p* = 0.03 for the maximal) and an increase in the skin blood flow disparity between fingers at the four points of laser speckle rewarming measurements (*p* = 0.03). After TSD, acute caffeine intake (vs. placebo) increased mean (+2.11°C ± 0.21°C, *p* = 0.01) and minimal (+0.61°C ± 0.10°C, *p* = 0.02) finger temperatures during CWI, and improved rewarming after CWI (mean and maximal temperatures) (+2.28°C ± 0.08°C, *p* = 0.01, and +2.06°C ± 0.12°C, *p* = 0.02, respectively). Before TSD, acute caffeine intake significantly increased (vs. placebo) mean temperatures during CWI (*p* = 0.03) and reduced pain from the onset (*p* = 0.03) to the end of CWI (*p* = 0.02) and the first 2 min of rewarming (*p* = 0.04). There was also a significant main effect of habitual daily caffeine consumption on minimal finger temperatures during CWI, which decreased significantly between 0 and 600 mg consumption (*R* ^2^ = −0.43, *p* = 0.01), independently of the effects of day (before and after TSD) and treatment (caffeine and placebo conditions). These findings suggest that acute caffeine intake could be a protective countermeasure to local cold tolerance, particularly during TSD. However, habitual daily caffeine consumption is a factor of individual variability that should be recorded during CWI protocols. Clinical trial NCT03859882.

## Abstract (highlights)

- **What is the central question of this study?**
  What is the effect of acute caffeine intake on local (finger) cold tolerance before and after total sleep deprivation (TSD)?
- **What is the main finding and its importance?**
  We found that acute caffeine intake (compared with placebo): (1) increased TSD‐related reductions in finger temperature and skin blood flow during rewarming after cold‐water immersion (CWI); and (2) decreased pain during CWI before TSD only. We also evidenced a significant effect of habitual daily caffeine consumption on minimal temperatures during CWI, without an interaction with TSD or acute caffeine intake.

## 1 INTRODUCTION

Cold stress is a common environmental constraint encountered by military personnel (Grieve et al., 2011; Moran et al., 2003), rescue teams (Küpper et al., 2003), climbers and indoor or outdoor workers in cold environments (Piedrahita et al., 2008). When exposed to cold, a decrease in peripheral temperatures, primarily skin, and core temperature elicits the primary cold thermoregulatory responses, that is, vasoconstriction of the skin to limit heat loss and shivering to produce more heat (Castellani & Young, 2016). Cutaneous vasoconstriction reduces skin blood flow (SkBF) in the peripheral limbs, particularly in the fingers, which are most likely to be exposed to cold directly. Therefore, the SkBF of the extremities can be almost negligible during exposure to extreme cold (Daanen, 2003; Kellogg Jr, 2006), and the skin can reach very low temperatures, increasing the risk of local cold injuries. However, cold exposure of the extremities (i.e., <15°C) might lead to a cold‐induced vasodilatation (CIVD), which occurs generally within the first 15 min of cold‐water exposure (Lewis, 1930). This vasodilatation is a cyclical, paradoxical increase in finger temperature (>1°C) on exposure to cold, which warms the extremities and protects the tissues (Cheung & Mekjavic, 2007; Daanen & Van Der Struijs, 2005). CIVD is usually studied during a 30 min cold‐water immersion (CWI) of the hand or finger, because it is a reproducible test (O'Brien, 2005), which has allowed evaluation of the microvascular reactivity of human skin (Sauvet et al., 2012; Sendowski et al., 1997). Recent data from military arctic training exercise suggested that finger CIVD might not be as predictive of cold injury in applied settings as once believed (Norrbrand et al., 2020; Sullivan‐Kwantes et al., 2019).

At the present time, the physiological mechanisms of the cutaneous vascular responses of the extremities to a local cold exposure are not well established. The main vasoconstrictor response of the human skin exposed to cold involves both reflexes and local factors (Kellogg Jr, 2006). In glabrous skin areas (fingers, palms and the plantar aspect of the feet), the role of sympathetic vasoconstrictor nerves in the initial phase of the local cooling response of the skin is well characterized (Hodges et al., 2018; Kellogg Jr, 2006). However, the non‐neural mechanisms involved in the prolonged response to local cooling, in particular those involved in CIVD, are not yet well described.

Sleep deprivation has been identified as an individual risk factor for changes in thermoregulatory function (Keramidas & Botonis, 2021) and cold injuries (Rintamäki, 2000). In our previous laboratory study, we demonstrated that total sleep deprivation (TSD) augments the reduction in finger temperature during CWI (i.e., the hand immersed in 5°C cold water for 30 min) and during rewarming in ambient air after CWI (i.e., rewarming for 30 min) (Sauvet et al., 2012). However, there was no significant effect of TSD on the number and characteristics of CIVD. In this study, we additionally demonstrated that the TSD‐related effects on cold tolerance (i.e., augmenting the finger temperature reduction during CWI and rewarming) could be related, in part, to higher levels of plasma endothelin after TSD, the most important vasoconstrictive agent produced by endothelial cells (Sauvet et al., 2012). That study in 2012 and others by our team have shown that TSD or sleep restriction (1 week) reduces local cold tolerance and endothelium‐dependent vasodilatation, independently of blood pressure and sympathetic activity (Sauvet et al., 2014, 2010, 2015).

Caffeine is used to counteract the sleep‐loss‐related neurobehavioural impairments (McLellan et al., 2016). It is a non‐selective antagonist of adenosine receptors (mainly A_1_ and A_2A_), which modulates glutamatergic, cholinergic, dopaminergic, serotoninergic and noradrenergic neurotransmission (Cappelletti et al., 2015). Caffeine has physiological effects on vascular muscle activity, heart rate (HR) and blood pressure, which depend on habitual consumption (Xu et al., 2021). Thus, most of the studies on the acute effect of caffeine reporting a blood pressure effect were performed in non‐habitual coffee drinkers or after a prolonged abstinence (Corti et al., 2002).

To provide nutritional information for people working in cold, high‐altitude environments, it was suggested in the 1990s that ephedrine/caffeine ingestion could represent a pharmacological tool for enhancing cold‐induced thermogenesis and delaying the onset of hypothermia in humans (Vallerand, 1993; Vallerand et al., 1989). Subsequently, Kim et al. (2013) investigated the effects of caffeine on the CIVD response to cold and suggested that caffeine intake (300 mg) negatively affects the protective mechanisms of the body's response to peripheral cold exposure because it decreases the mean finger temperature during CWI. Another study showed that caffeine ingestion (250 mg) decreased the level of pain during the cold pressor task, that is, hand in an ice‐cold‐water bath maintained at a temperature between 0°C and 3°C, in healthy normotensive men (Keogh & Witt, 2001). To our knowledge, there is no information on the effects of habitual caffeine consumption on tolerance to local cold in sleep‐deprived subjects.

We hypothesize that acute caffeine intake might increase the negative effect of sleep deprivation on local cold tolerance, possibly through its effects on the sympathetic and vascular systems (Corti et al., 2002; Xu et al., 2021). Therefore, the main purpose of this study was to characterize the effects of acute caffeine intake on tolerance to local cold exposure before and after a period of 33 h of TSD in the laboratory. The local cold tolerance was evaluated through changes of the following: (1) finger temperature during a CWI test followed by rewarming in ambient air temperature; (2) SkBF during rewarming to ambient air temperature after CWI; and (3) pain levels. Given that many studies concerning acute caffeine effects have been made after a period of caffeine withdrawal (Hansen et al., 2019) or considering habitual caffeine consumption as an exclusion criterion (Kim et al., 2013), which does not reflect real‐life conditions in general, we chose not to require caffeine withdrawal and considered habitual daily caffeine consumption as a covariable in our statistical analyses.

## 2 MATERIALS AND METHODS

### 2.1 Subjects

Thirty‐eight subjects, aged between 22 and 52 years, were included. The study received the agreement of the Cochin—CPP Ile de France IV (Paris) Ethics Committee and of the French National Agency for Medicines and Health Products Safety (ANSM) (Ile de France IV) (ID‐RCB: 2017‐A02793‐50), and was conducted according to the principles expressed in the *Declaration of Helsinki* of 1975, as revised in 2004. All the subjects gave their informed written consent. This study was part of a fairly extensive laboratory protocol, devoted in its primary outcome to the effects of caffeine on cognitive performance during TSD in healthy subjects. This work is a part of a protocol registered in the clinical trials database (NCT03859882).

All subjects gave a detailed medical history and underwent a full medical examination. Exclusion criteria were as follows: presence of a history of cold injuries and vascular pathologies, workers subject to frequent occupational cold exposure, shift‐workers, smokers, daily consumption of alcoholic beverages and those consuming >500 mg of caffeine per day, subjects with a body mass index of >30 kg/m^2^, and those taking medication (Daanen, 2003; Ooijen et al., 2006). Subjects with excessive daytime somnolence (Epworth Sleepiness Scales ≥ 9) (Johns, 1992) or sleep complaints (Pittsburg sleep quality index > 8) (Buysse et al., 1989) were also excluded, as were any volunteers considered to have an extreme morning‐to‐evening chronotype (score < 31 and > 69) according to the Horne and Ostberg questionnaire (Horne & Ostberg, 1976). Sleep/wake patterns were checked using wrist actigraphy (Actiwatch™; Cambridge Neurotechnology, Cambridgeshire, UK) 1 week before the experiment.

Of the 38 subjects who took part in this study, two were excluded, the first because of a significant adverse effect after caffeine intake (nausea and vomiting, this is a person [she] who never drank coffee or tea because she did not like the taste) and the second because of a very high pain level (10/10) and unacceptable suffering during the CWI test. Finally, a total of 36 healthy subjects (33.5 ± 7.8 years) completed the protocol, including 20 women and 16 men.

### 2.2 Categorization of habitual caffeine consumption

After inclusion, subjects completed a questionnaire including the following beverages and caffeine‐containing foods: coffee with caffeine, tea, cola and other carbonated beverages with caffeine, and chocolate. For each item, participants were asked how often, on average, they had consumed a specified amount of each beverage or food over the past year. The participants could choose from nine frequency categories (never, 1–3 per month, 1 per week, 2–4 per week, 5–6 per week, 1 per day, 2–3 per day, 4–5 per day, and 6 or more per day). Typical milligram doses (Mayo Clinic; http://www.mayoclinic.com/health/caffeine/AN01211) were assigned to each, and an approximate daily consumption was obtained.

### 2.3 Study design and testing conditions

In a crossover laboratory protocol, we first evaluated influences of acute caffeine (vs. placebo) intake on local (fingers) cold tolerance before and after 33 h of continuous wakefulness (i.e., TSD). Secondly, the influence of habitual daily caffeine consumption was examined. This study has been conducted in the Armed Forces Biomedical Research Institute (IRBA), Brétigny sur Orge, France. The subjects came to the laboratory twice. The two laboratory periods were separated by 2 weeks.

**FIGURE 1:**
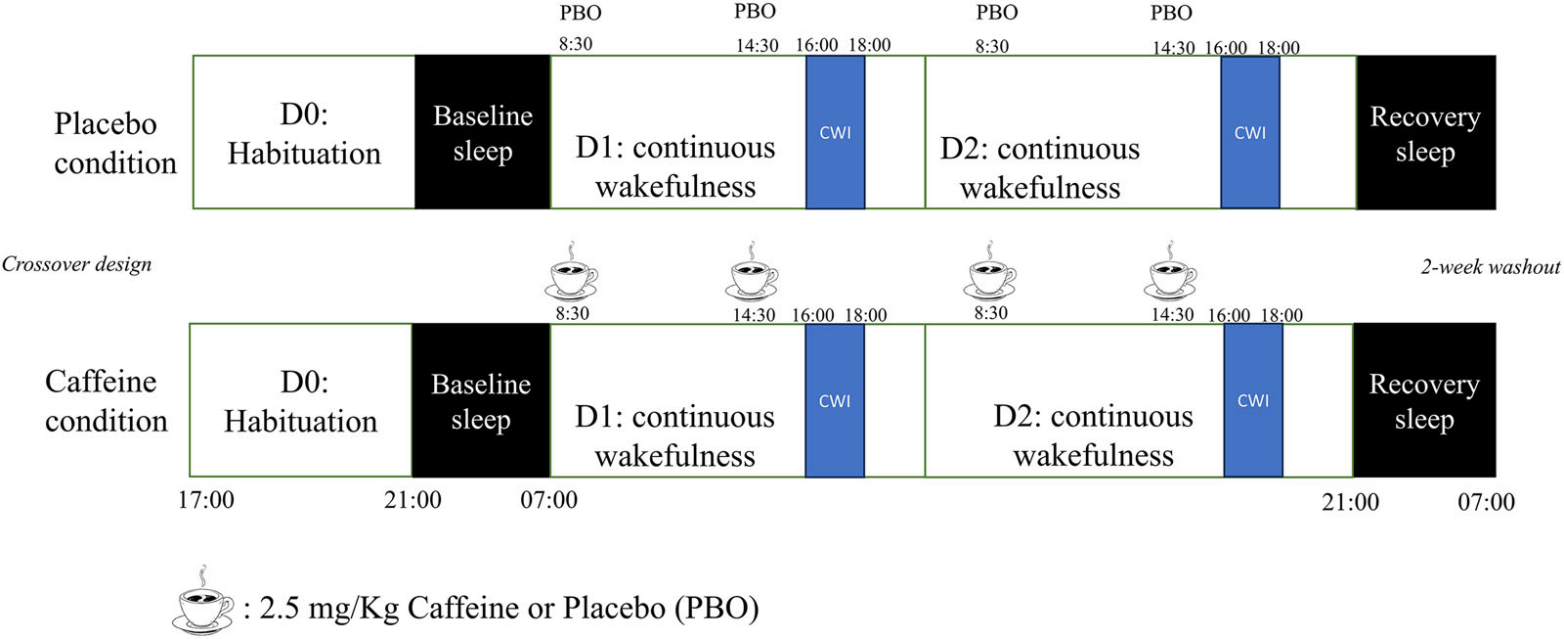
Study design. CWI, cold‐water immersion; PBO, placebo.

For each period, subjects remained inside the laboratory for 3 days consecutively. The 3 day in‐laboratory experimental protocol included: (1) a habituation day (D0; to familiarize subjects with the laboratory environment and the tests, in order to avoid a learning or surprise effect); (2) a baseline day (D1); (3) a TSD day beginning on D1 at 07.00 h until D2 at 21.00 h (meaning 38 h of continuous wakefulness); and (4) a recovery night before leaving the laboratory at 09.00 h (Figure 1). Ambient temperature was controlled and maintained at 22°C ± 1°C throughout the experiment. The brightness of the lighting was maintained between 150 and 200 lx during the awake periods, and lights were off during sleep periods. Meals and caloric intake were standardized for all subjects (2600 kcal/day, including 15%–20% proteins and 40%–50% carbohydrates), in line with the recommendations of ANSES (the French Agency for Food, Environmental and Occupational Health and Safety). Subjects were asked to maintain their habitual caffeine consumption during the week before the experiment. In the laboratory, they were not allowed to practise exercise, taking tobacco, alcohol or other psychoactive substances during the study. We did not ask the volunteers to stop eating foods such as grapefruit or orange juice, which contain flavonoids (these can act, if taken as supplements, on caffeine metabolism). Subjects were under visual surveillance by research staff members, and we used wrist actigraphy to check that the subjects stayed awake during the 38 h continuous wakefulness period. When they were not engaged in testing, meals or sleep periods, participants were allowed to read, to watch videos or to speak with other participants or staff members and play games, following a pre‐established programme.

### 2.4 Caffeine and placebo intake

Caffeine or placebo (PBO) was administered in a decaffeinated beverage. In the caffeine condition, each participant received 2.5 mg/kg body weight of caffeine powder mixed in a decaffeinated beverage. The placebo was the decaffeinated beverage. In order to design a placebo that could not be differentiated from the beverage containing the caffeine (smell, taste, colour and bitterness), we used an intensity 9 Nespresso coffee (Nestlé SA, Vevey, Switzerland). The caffeine powder was added to an intensity 3 Nespresso coffee. We have previously verified that six blinded members of the staff (three successive tests) were unable to distinguish the drink containing caffeine from the placebo (10 errors out of 18 tests). We also checked that the decaffeinated drink did not contain caffeine. The caffeine powders were pre‐measured by the project supervisor.

This amount of caffeine powder was chosen for its enhancing properties on attention in sleep‐deprived conditions (2.5–8 mg/kg of caffeine) (McLellan et al., 2016). The beverage was administered at 08.30 and 14.30 h (after 1.5 and 7.5 h of prolonged wakefulness) on D1, and at 08.30 and 14.30 h (after 25.5 and 31.5 h of prolonged wakefulness) on D2.

### 2.5 Finger CWI test

Each subject completed two CWI tests at 16.00 h: one on D1, after the first night with habitual sleep (i.e., 8 h time in bed) (i.e., before TSD), and one at D2, after 33 h of wakefulness considered as TSD (i.e., after TSD). Prior to the test, subjects were familiarized with the instruments, body position, chamber environment and the water temperatures used for testing. During the tests they wore standardized clothing: shorts, T‐shirt, shoes and socks. They were asked to minimize movement and conversation during the tests.

**FIGURE 2:**
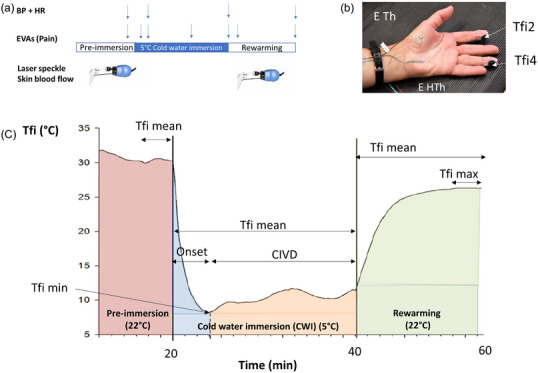
(a) Experimental protocol. (b) Instrumentation of the immersed hand and localization of hand and fingers 2 (F2) and 4 (F4) measures of cutaneous temperature. (c) Cutaneous finger temperature parameters calculated during the pre‐immersion period of the CWI test, and during immersion and rewarming. Pre‐immersion is considered as the last 5 min of the 20 min pre‐immersion period. CWI is the 20 min of CWI (5°C). Rewarming is the 20 min period in ambient air after CWI. Onset is the initial minimal finger (Tfi) temperature during CWI. Abbreviations: BP, blood pressure; CIVD, cold‐induced vasodilatation; CWI, cold‐water immersion; EVAs, visual analog scale; HR, heart rate.

The CWI test was carried out in a climate‐controlled room (temperature = 22°C ± 2°C; relative humidity = 30%–50%). After entering the room, the subject was instrumented and sat for 20 min with the hand in the air (pre‐immersion). The subject then immersed his/her fingers (fingers 2–5), up to the metacarpal tips, in a cold‐water bath maintained at 5°C (Ministrat 125; Huber, Offenburg, Germany) for 20 min. At the end of the CWI, the subject removed his/her hand from the water bath and dried it with a towel before the 20 min rewarming period began (Figure 2a,c).

### 2.6 Instrumentation

During the experiment, the skin temperatures of the fingers 2 (Tfi2) and 4 (Tfi4), of the thenar and hypothenar eminences (E Th and E HTh, respectively) of the right hand and the temperature of the middle of the right arm (Tarm) were recorded continuously using copper–constantan (Cu–Ct) thermocouples insulated with a small patch of Neoprene (Figure 2b). We used adhesive tape (Blenderm, 3M, Saint Paul, MN, USA) to secure the patch to ensure that no water penetrated between the skin and the thermocouple. All the thermocouples were previously calibrated using a high‐precision thermometer (±0.01°C; PHP602 thermometer with PT100‐5847‐03 probe and a Hyperion‐Basic calibration bath; AOIP, Ris Orangis, France). Body core temperature (BCT) was also recorded continuously using an ingested temperature‐monitoring capsule (BodyCap e‐Celsius; BodyCap, Hérouville‐Saint‐Clair, France).

During the pre‐immersion and rewarming periods, SkBF in the right arm was measured using laser speckle contrast analysis (LASCA) (Pericam, Perimed, Jarfalla, Sweden) (Figure 2a). This is a safe technique to quantify blood perfusion at different regions of interest (ROIs). Five ROIs were created in the central area of the fingertips, from the second to the fifth finger, and in the palm of the immersed hand. The SkBF was recorded in perfusion units (PU) (Cracowski et al., 2011).

Systolic (SAP) and diastolic (DAP) arterial blood pressures were measured every minute from the left arm using an automatic sphygmomanometer, and HR was monitored continuously by a HR monitor (Dinamap PLUS, model 8723; Critikon, USA).

### 2.7 Subjective pain assessment

During the test, the volunteers were asked to rate their pain using a continuous pain visual analogue scale (VAS) ranging from 0 (‘no pain at all’) to 10 (‘unbearable pain’) at the end of the pre‐immersion period, after 2 min of CWI, during CIVD, at the end of the CWI, after 2 min of rewarming and at the end of the rewarming (Figure 2a).

### 2.8 Calculations

For each subject, mean temperature values per minute (core and skin) were calculated. The BCT, HR and blood pressure variables were calculated for the last 5 min of the 20  min pre‐immersion period, for the 20 min CWI period and for the last 5 min of the 20 min rewarming period. Finger temperature data were smoothed by using a three‐point running average to reduce noise, and a transitory change in temperature of ≥0.5°C was chosen to represent an occurrence of CIVD (Mekjavic et al., 2013; O'Brien, 2005).

**FIGURE 3:**
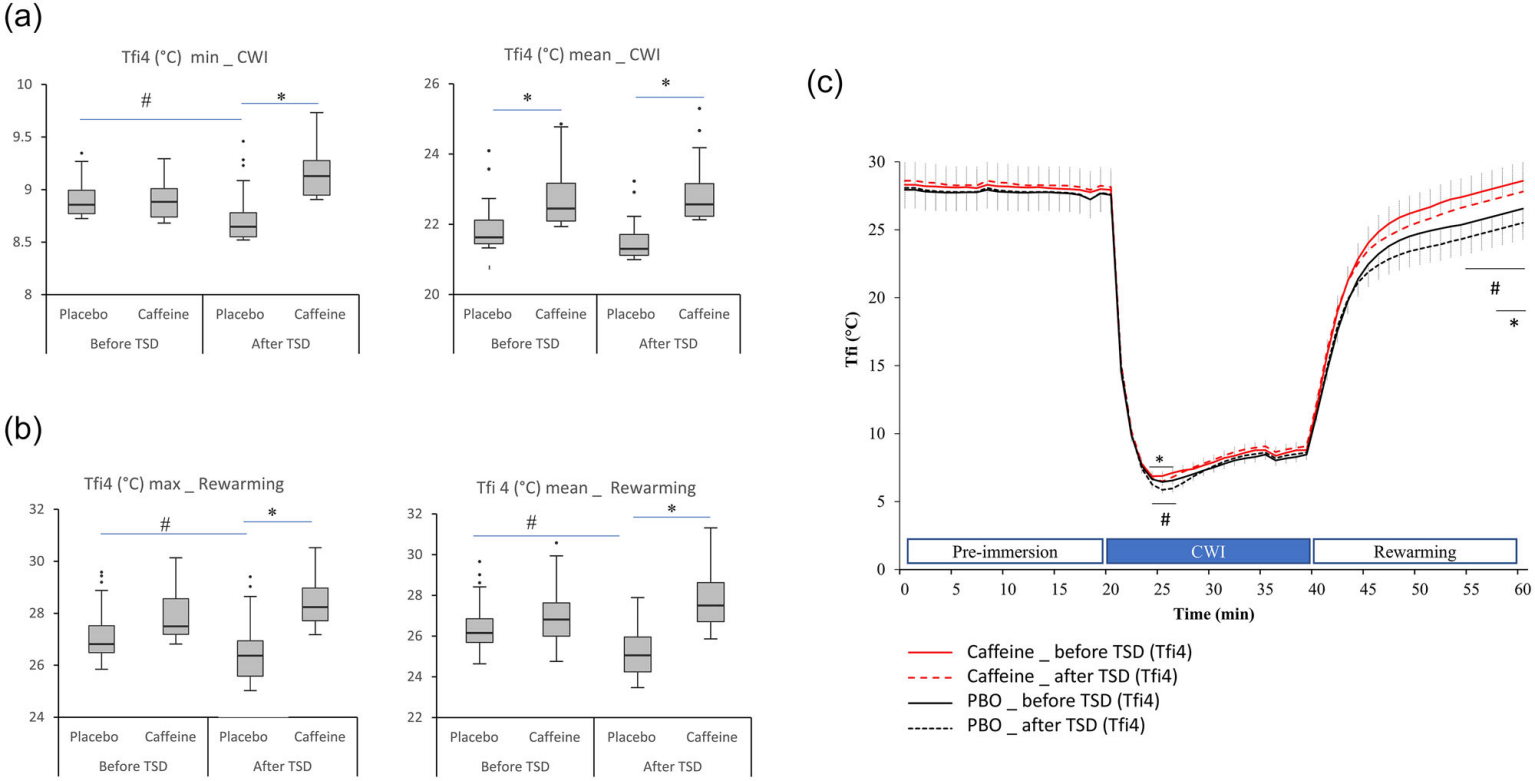
(a, b) Box‐and‐whisker plots for changes in finger 4 temperatures (Tfi4), minimal (min) and mean, during CWI (a) and maximal (max) and mean during rewarming (b) before and after TSD in the caffeine and PBO conditions. The box encompasses the 25%–75% quartiles, and the median is represented by the horizontal line within the box. The whiskers extend to the highest and lowest values. (c) The averaged raw data before TSD and after TSD in the placebo and caffeine conditions. ^*^ *p *< 0.05, difference between PBO and caffeine conditions; ^#^ *p *< 0.05, difference between before and after TSD. Values are means ± SD, and *n* = 36. Abbreviations: CWI, cold‐water immersion; PBO, placebo; TSD, total sleep deprivation; Tfi4, finger 4 temperature.

Finger temperatures (Figure 2c) were assessed by examining the following variables across the CWI: minimal (min) and mean Tfi values (Tfi mean and Tfi min CWI) (Sendowski et al., 1997). During rewarming, we calculated the TFi maximal (max) and the mean values during the whole rewarming period (Tfi mean and Tfi max rewarming) (Figure 3).

**FIGURE 4:**
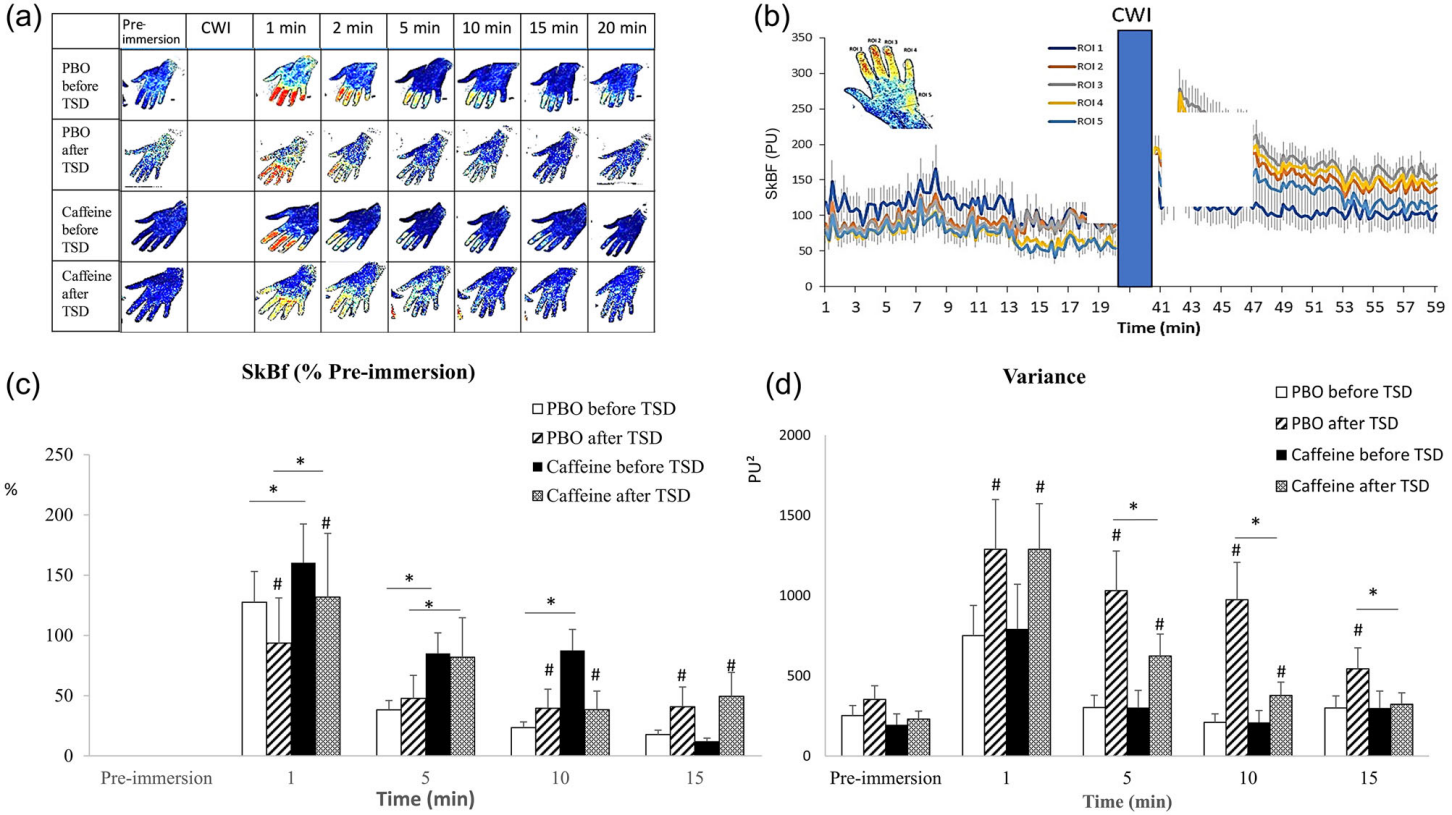
(a) Finger ROI images assessed by the laser speckle imager, for one subject, at pre‐immersion and at all five measurement points during rewarming after CWI in PBO and caffeine conditions and before and after TSD. (b) The SkBF means in each ROI for all subjects (before TSD and in the placebo condition) during the pre‐immersion and rewarming periods after CWI (each colour represents one ROI). (c) Cutaneous SkBF during pre‐immersion and the 15 min of rewarming after CWI before and after TSD, in the placebo and caffeine conditions (means values are expressed as a percentage of changes from the pre‐immersion mean values). (d) Variance of SkBF changes between ROIs during the pre‐immersion and the 15 min of rewarming after CWI, in the placebo and caffeine conditions. ^*^ *p *< 0.05, difference between PBO and caffeine conditions; ^#^ *p *< 0.05, difference between before and after TSD. Values are means ± SD, and *n* = 36. Abbreviations: CWI, cold‐water immersion; PBO, placebo; ROI, regions of interest; SkBF, skin blood flow; TSD, total sleep deprivation.

The SkBF signal (expressed in arbitrary perfusion units) from the laser speckle was recorded every 10 s (Cracowski et al., 2011) (Figure 4a). For each ROI (Figure 4b), the SkBF recovery rate at the nail fold of each finger was calculated as the SkBF value divided by the pre‐immersion value (i.e., the data are normalized because of high inter‐individual variability, and results are provided as the percentage change from pre‐immersion; Figure 4c). The SkBF disparity between the five fingers was calculated as the variance of SkBF changes (Figure 4d) (Bland, 2015).

Mean arterial pressure (MAP; in millimetres of mercury) was calculated as: MAP = SAP/3 + (2/3)DAP.

### 2.9 Statistical analyses

Statistical analyses were computed using Jamovi for R [v.1.6, the Jamovi project (2021), retrieved from https://www.jamovi.org]. Values were expressed as the mean ± SD. The significance level has been fixed under 5%. Firstly, for all parameters, we performed a mixed linear model with two fixed factors, treatment (caffeine vs. PBO) and day (after TSD vs. before), and with the habitual caffeine consumption as a covariable continuous factor. Subjects were considered as a random factor for treatment and day. Degrees of freedom have been calculated using the Satterthwaite method. This was followed by Tukey's *t*‐test to compare parameters between caffeine versus PBO conditions, and between before versus afer TSD. Secondly, we assessed Pearson's correlation coefficients between habitual caffeine consumption and temperature values during immersion and rewarming.

## 3 RESULTS

Subjects’ average daily caffeine consumption was 250 ± 182 mg. The average weight was 67.5 ± 3.6 kg and the average body mass index was 23.5 ± 3.6 mg/m^2^. The mean weekly exercise duration was 3.1 ± 2.4 h. The mean daily total sleep time was 7.2 ± 1.2 h, and the Epworth sleepiness score was 6.8 ± 3.6. The chronotypes were distributed as follows: 19 subjects were of intermediate chronotype, 16 were of the morning chronotype, and 1 was of the evening chronotype. We checked that laboratory and cold bath temperatures were well controlled, without a significant difference related to day (after vs. before TSD) or treatment (PBO vs. caffeine) effects.

During the pre‐immersion period, the average BCT was 37.3 ± 0.6°C in the placebo condition, and before TSD, the E Th temperature was 30.0 ± 2.5°C, the E HTh temperature was 30.4 ± 2.4°C (Table A1), the laboratory temperature was 22.7± 1.3°C, and the bath temperature was 5.1 ± 0.2°C. During the pre‐immersion period CWI, we observed no significant effect of the day (before vs. after TSD) or treatment (caffeine vs. PBO), or interaction, on skin of fingers 2 and 4 (Tfi2 and Tfi4) and E Th and E HTh temperatures (Table A2). Pre‐immersion temperatures for Tfi2 and Tfi4 were 27.3 ± 2.4°C and 26.8 ± 2.6°C, respectively.

During the CWI, we observed no significant effect of the day or treatment, or interaction, on BCT (Table A3). Compared with pre‐immersion, E Th and E HTh temperatures were significantly lower before and after TSD, and in both caffeine and placebo conditions (Table A1). During the CWI onset, we observed an expected decrease of the finger temperatures (Tfi; i.e., compared with pre‐immersion; for greater clarity, only changes of Tfi4 are shown in Figure 3c). The lower temperatures have been observed at the onset point (Tfi2 and Tfi4 were 8.75 ± 2.40°C and 8.86 ± 2.40°C, respectively).

During the CWI, we observed one CIVD wave in 32 subjects, with no significant effect of day or treatment. A transient CIVD wave was not observed in four subjects; in them, we observed a continuous progressive increase in finger temperature throughout CWI. Significant effects of treatment were observed for the mean and min Tfi2 and Tfi4 temperatures, and significant interaction with the day main effect only for min temperatures (Table 1). There was a lower min temperature after TSD in the placebo condition (*p* = 0.02). After TSD, the mean and min temperatures were significantly higher in the caffeine compared with the placebo condition (mean, +2.1°C ± 1.5°C for Tfi2, *p* = 0.01, and +1.3°C ± 1.6°C for Tfi4, *p* = 0.01; min: +0.68°C ± 0.15°C for Tfi2, *p* = 0.03, and +0.61°C ± 0.14°C for Tfi4, *p* = 0.03; Tfi4 values are shown in Figure 3a, c). Moreover, during CWI, we observed a significant effect of habitual daily caffeine consumption only for min Tfi2 and Tfi4 temperatures (Table 1), and a negative correlation is present between the two variables (*R* ^2^ = −0.30, *p* = 0.02 for Tfi2; and *R* ^2^ = −0.43, *p* = 0.01 for Tfi4). No significant interaction was observed between habitual daily caffeine consumption and the day and treatment effects (Table 1). No significant day and treatment effects were observed for arm temperature (Th or HTh areas) during CWI (Table A2).

**TABLE 1:** Statistical results of the mixed model during cold‐water immersion for finger skin temperature.

			Finger 2		Finger 4	
Parameter	Effects	d.f.	*F*	*p*‐value	*F*	*p*‐value
Mean	Day	1	1.16	0.28	0.23	0.63
	Treatment	**1**	**8.16**	**0.01**	**13.33**	**0.01**
	Caffeine consumption	2	2.76	0.04	2.37	0.68
	Day × Treatment	1	1.53	0.79	0.37	0.54
	Day × Caffeine consumption	2	0.60	0.23	1.16	0.32
	Treatment × Caffeine consumption	2	1.54	0.08	1.16	0.09
	Day × Treatment × Caffeine consumption	2	0.22	0.83	1.11	0.33
Minimum	Day	1	0.16	0.97	1.15	0.29
	Treatment	**1**	**3.43**	**0.03**	**4.20**	**0.02**
	Caffeine consumption	**2**	**2.09**	**0.03**	**3.16**	**0.03**
	Day × Treatment	**1**	**2.21**	**0.08**	**3.47**	**0.04**
	Day × Caffeine consumption	2	1.09	0.16	1.16	0.12
	Treatment × Caffeine consumption	2	1.59	0.09	1.16	0.10
	Day × Treatment × Caffeine consumption	2	0.11	0.34	1.30	0.28

*Note*: ‘Minimum’ is the minimal temperature during cold‐water immersion. ‘Day’ is after total sleep deprivation versus before. ‘Treatment’ is caffeine condition versus placebo. ‘Caffeine consumption’ is the habitual daily caffeine consumption. Values of *p* < 0.05 are in bold.

During the 20 min rewarming period after CWI, we observed no significant effect of the day or treatment or interaction on BCT (Table A4). Compared with pre‐immersion, E Th and E HTh temperatures were significantly lower before and after TSD and in both caffeine and placebo conditions (Table A1). Regarding the finger 2 and 4 temperatures, we observed significant effects of treatment and day for the max temperatures (Table 2). A significant treatment effect was observed for the mean temperatures, with significant interaction with the day effect. In the placebo condition, there was lower mean and max temperatures of the two fingers (Tfi2 and Tfi4) after TSD compared with before (*p* = 0.03); this difference was not observed in the caffeine condition (*p* = 0.22) (Tfi4 values are shown in Figure 3b,c). After TSD, the mean and max temperatures were significantly higher in the caffeine compared with the placebo condition (mean, +2.12°C ± 0.20°C for Tfi2, *p* = 0.02, and +2.28°C ± 0.23°C for Tfi4, *p* = 0.03; max, +2.05°C ± 0.21°C for Tfi2, *p* = 0.03, and +2.08°C ± 0.25°C for Tfi4, *p* = 0.03; Tfi4 values are shown in Figure 3b,c). The treatment effect on the mean temperatures was present over the last 4 min of the 20 min rewarming period (shown in Figure 3c). Furthermore, no significant effect of habitual daily caffeine consumption was observed for maximal and mean temperatures, nor any interaction with day and treatment (Table 2).

**TABLE 2:** Statistical results of the mixed model during rewarming after cold‐water immersion for finger skin temperature.

			Finger 2		Finger 4	
Parameter	Effects	d.f.	*F*	*p*‐value	*F*	*p*‐value
Mean	Day	1	0.23	1.15	1.15	0.28
	Treatment	1	**9.30**	**0.01**	**12.54**	**0.001**
	Caffeine consumption	2	0.37	0.79	0.79	0.46
	Day × Treatment	1	**2.97**	**0.04**	**2.61**	**0.04**
	Day × Caffeine consumption	2	0.16	0.59	0.59	0.55
	Treatment × Caffeine consumption	2	1.16	0.12	1.23	0.13
	Day × Treatment × Caffeine consumption	2	1.11	0.17	0.17	0.83
Maximum	Day	1	**2.05**	**0.02**	**2.18**	**0.02**
	Treatment	1	**12.02**	**0.001**	**13.54**	**0.001**
	Caffeine consumption	2	0.36	0.34	0.34	0.70
	Day × Treatment	1	0.29	0.14	0.14	0.70
	Day × Caffeine consumption	2	1.86	0.19	0.19	0.82
	Treatment × Caffeine consumption	2	1.40	1.28	1.28	0.28
	Day × Treatment × Caffeine consumption	2	0.33	1.50	1.50	0.23

*Note*: ‘Minimum’ is the minimal temperature during cold‐water immersion. ‘Day’ is after total sleep deprivation versus before. ‘Treatment’ is caffeine condition versus placebo. ‘Caffeine consumption’ is the habitual daily caffeine consumption. Values of *p* < 0.05 are in bold.

In the statistical analysis, we also observed that the mean rewarming temperatures of fingers remained lower than pre‐immersion (before CWI) after TSD (24.5 ± 5.1°C) when compared with before TSD (25.6 ± 4.8°C) in the PBO condition, with a mean difference of 1.2 ± 0.2°C and 1.3°C ± 0.3°C for Tfi2 and Tfi4 (*p* = 0.04). This was not observed in the caffeine condition, with fingers temperatures being not different from the pre‐immersion values at the end of the rewarming period (for Tfi2, 27.3 ± 2.4°C at pre‐immersion and 27.1 ± 2.4°C at rewarming, *p* = 0.87).

**FIGURE A1:**
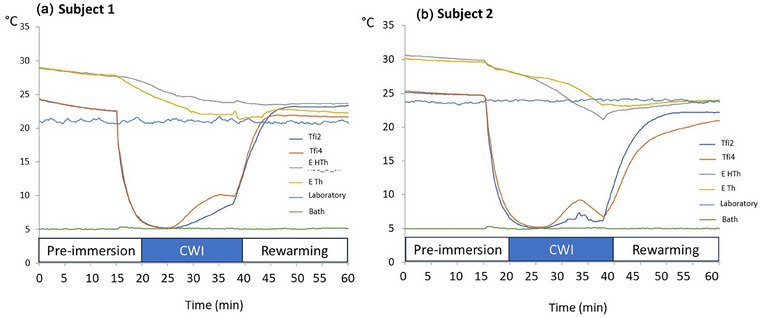
Example for two subjects (a, b) of temperature patterns of fingers 2 and 4 (Tfi2 and Tfi4), thenar and hypothenar eminence temperature (E Hth and E Th), laboratory ambient temperature and bath temperature during the cold immersion test. (a) We observed a progressive increase of finger temperature during CWI. (b) A classic CIVD response during CWI. Abbreviations: CIVD, cold‐induced vasodilatation; CWI, cold‐water immersion.

Finally, Figure A1 in the Appendix shows examples (two subjects) of skin temperature responses of fingers 2 and 4, before TSD and in the placebo condition, including E Th and E HTh responses, laboratory ambient temperature, and bath temperature during the cold immersion test.

Regarding laser speckle perfusion (SkBF) and variance values of the four finger ROIs during the 20 min rewarming period, we observed significant effects of day and treatment (otherwise larger on variance), without interaction (Table 3). In the PBO condition, SkBF was lower after TSD after (1 min) removing the fingers from cold water and higher after 10 and 15 min of rewarming; variance remained higher for all four time measurements (Figure 4c,d). In the caffeine condition, there was lower SkBF after TSD after (1 min) removing the fingers from cold water and at 10 min of rewarming; the variance remained higher at the first three measurement points (Figure 4c,d). Caffeine treatment significantly increased SkBF before TSD (first three measurements) and after TSD (first two measurements), whereas it decreased variance only after TSD at 5, 10 and 15 min of rewarming (Figure 4c,d). We observed that the lowering effect of TSD on SkBF was present in the first 5 min of rewarming (*p* = 0.02; Figure 4c). Finally, there was no significant effect of habitual daily caffeine consumption or interaction with day and treatment for SkBF and the variance (Table 3). Figure 4a shows finger ROI images for one subject at pre‐immersion and at all five measurement points during rewarming after CWI in the PBO and caffeine conditions and before and after TSD. Figure 4b shows SkBF changes in each ROI for one subject during the pre‐immersion and rewarming after CWI periods.

**TABLE 3:** Statistical results of the mixed model during rewarming after cold‐water immersion for skin blood flow values.

		Mean skin blood flow		Variance	
Effects	d.f.	*F*	*p*‐value	*F*	*p*‐value
Day	1	**3.16**	**0.04**	**6.16**	**0.01**
Treatment	1	**6.01**	**0.02**	**12.16**	**0.001**
Caffeine consumption	2	0.56	0.36	0.94	0.16
Day × Treatment	1	1.23	0.39	0.21	0.32
Day × Caffeine consumption	2	0.70	0.25	1.09	0.16
Treatment × Caffeine consumption	2	2.42	0.08	2.51	0.06
Day × Treatment × Caffeine consumption	2	0.21	0.24	1.40	0.29

*Note*: ‘Day’ is after total sleep deprivation versus before. ‘Treatment’ is caffeine condition versus placebo. ‘Caffeine consumption’ is the habitual daily caffeine consumption. Values of *p* < 0.05 are in bold.

**FIGURE 5:**
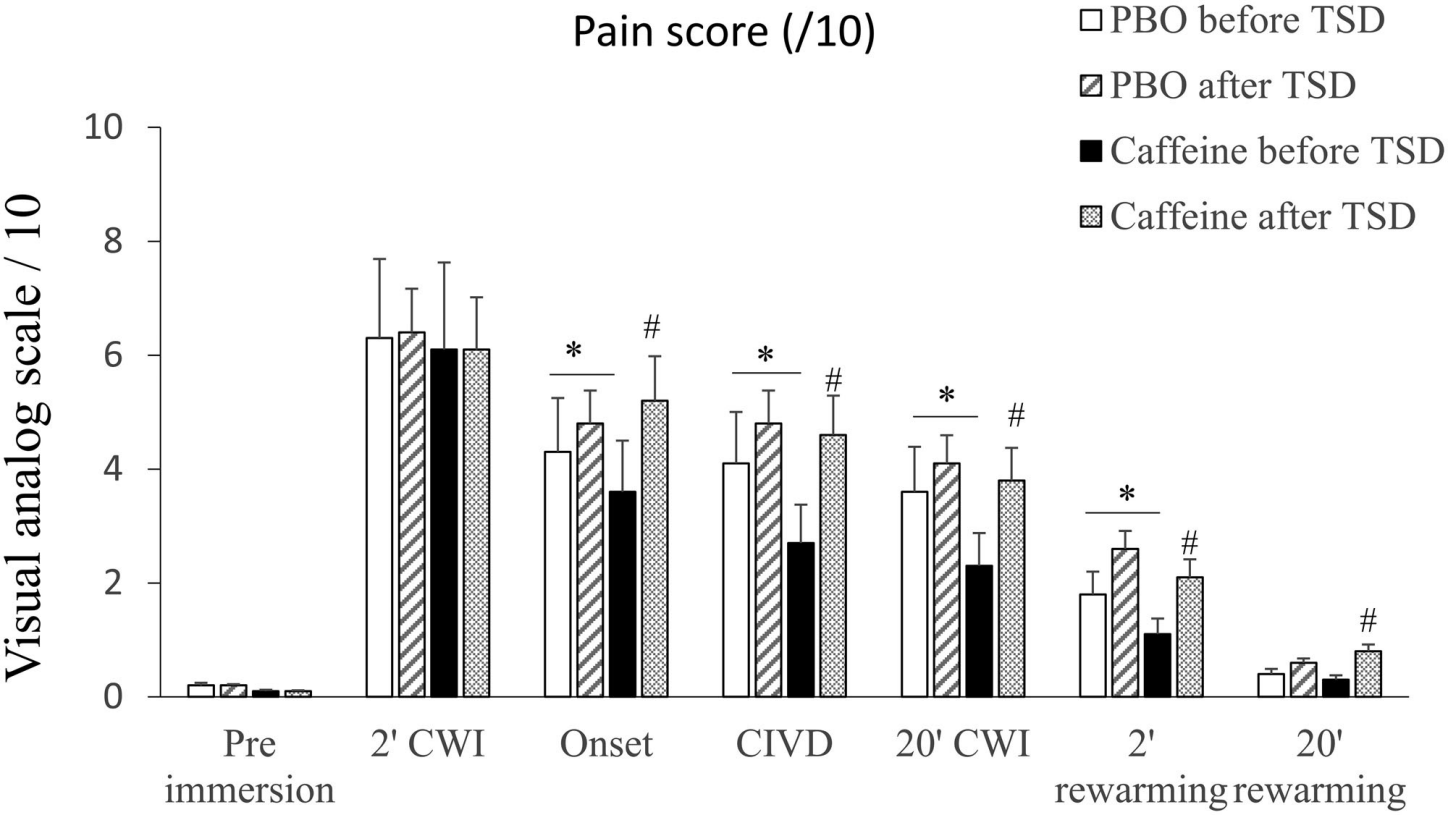
Evolution of pain (visual analog scales) during pre‐immersion before the CWI test, after 2 min (2′) CWI, 20 min (20′) CWI (including onset, CIVD and end of CWI), and after 2 min (2′) and 20 min (20′) of rewarming, in the PBO and caffeine conditions. ^*^ *p *< 0.05, difference between PBO and caffeine conditions; ^#^ *p *< 0.05, difference between before and after TSD. Values are means ± SD, and *n* = 36 except for CIVD, where *n* = 32, because four subjects did not have CIVD. Abbreviations: CIVD, cold‐induced vasodilatation; CWI, cold‐water immersion; PBO, placebo; ROI, regions of interest; TSD, total sleep deprivation.

Regarding pain values, we observed a significant day effect at the onset, CIVD, end of CWI and 2 min of rewarming, with a significant interaction between day and treatment for CIVD, end of CWI and 2 and 20 min rewarming (Table 4). There was greater pain after TSD in the caffeine condition from the onset of CWI (*p* = 0.001) to the end of rewarming (20 min duration) [CIVD, *p* = 0.001; end CWI (20 min), *p* = 0.001; 2 min rewarming, *p* = 0.02; 20 min rewarming, *p* = 0.03; Figure 5]. The pain was lower in the PBO condition from the onset of CWI (*p* = 0.03) to 2 min of rewarming [CIVD, *p* = 0.02; end of CWI (20 min), *p* = 0.03; 2 min rewarming, *p* = 0.04; Figure 5]. No effect of caffeine was observed after TSD at 2 min after CWI (*p* = 0.12; Figure 5). Finally, there was no significant effect of habitual daily caffeine consumption, nor interaction with day or treatment (Table 4).

**TABLE 4:** Mixed effect values for pain assessed at pre‐immersion, during cold‐water immersion and during rewarming after cold‐water immersion.

		2 min CWI		Onset		CIVD		End CWI		2 min rewarming		20 min rewarming	
Effects	d.f.	*F*	*p*‐value	*F*	*p*‐value	*F*	*p*‐value	*F*	*p*‐value	*F*	*p*‐value	*F*	*p*‐value
Day	1	0.08	0.96	**9.44**	**0.01**	**8.10**	**0.01**	**5.13**	**0.03**	**2.21**	**0.04**	1.23	0.12
Treatment	1	1.48	0.24	0.50	0.48	1.77	0.19	1.23	0.28	1.13	0.36	1.76	0.19
Caffeine consumption	2	2.07	0.13	0.83	0.49	0.46	0.70	0.57	0.63	0.67	0.62	0.34	0.72
Day × Treatment	1	0.39	0.52	1.00	0.32	**2.53**	**0.02**	**4.91**	**0.01**	**3.22**	**0.03**	**4.75**	**0.03**
Day × Caffeine consumption	2	0.23	0.85	0.37	0.77	0.53	0.66	0.93	0.44	0.54	0.66	0.43	0.32
Treatment × Caffeine consumption	2	0.30	0.84	1.04	0.39	0.51	0.67	0.31	0.81	0.42	0.68	1.06	0.40
Day × Treatment × Caffeine consumption	2	0.29	0.87	1.37	0.26	0.58	0.62	0.58	0.63	0.26	0.83	1.38	0.26

*Note*: ‘Day’ is after total sleep deprivation versus before. ‘Treatment’ is caffeine condition versus placebo. ‘Caffeine consumption’ is the habitual daily caffeine consumption. Values of *p* < 0.05 are in bold.

Abbreviations: CIVD, cold‐induced vasodilatation; CWI, cold‐water immersion.

During the CWI and in comparison to the pre‐immersion period, we observed significant increases in the placebo condition of the SAP (116.2 ± 18.5 vs. 134.9 ± 15.5 mmHg, *p* = 0.002), the DAP (68.3 ± 6.4 vs. 82.9 ± 12.4 mmHg, *p* = 0.02), the MAP (84.2 ± 18.9 vs. 94.2 ± 15.3 mmHg, *p* = 0.03) and HR (77.8 ± 19.2 vs. 91.2 ± 12.4 beats/min, *p* = 0.02) (Table A1). Furthermore, we observed no significant effects of day, treatment or habitual daily caffeine consumption during pre‐immersion, during CWI (except a treatment effect for HR at *p* = 0.05) and during rewarming (Tables A2, A3, A4).

## 4 DISCUSSION

In this work, we partly confirmed our previous study showing that TSD decreases fingers rewarming (temperatures and SkBF) in the CWI test in healthy subjects (Sauvet et al., 2012). In addition, we showed that acute intake of a moderate dose of caffeine (350 mg/day for a 70 kg person, referred to as the treatment factor in the statistical analysis) increases finger cold tolerance and limits the effects of TSD. Habitual daily caffeine consumption appears to interact with this response, because it is negatively correlated with minimal finger temperature, the latter being lower the higher the daily consumption. During the rewarming period after CWI, there is also an increase of variance of the SkBF between fingers after sleep deprivation.

In this study, conducted in controlled laboratory conditions, we were able to test the influence of TSD on local cold tolerance using the CWI test in a larger number of subjects than in our previous study (Sauvet et al., 2012). We confirm that TSD decreases the mean temperatures particularly during passive rewarming to ambient air (Sauvet et al., 2012). In a study conducted on military personnel working in a cold region, it was observed that subjects with slower recovery (i.e., lower finger temperature during passive rewarming) after 10 min of immersion in 10°C water had a higher incidence of cold‐related injuries during military exercises (Brändström et al., 2008). However, this has not been confirmed by other studies (Sullivan‐Kwantes et al., 2019), hence we can only suggest that the observed delay in rewarming could lead to a higher risk of cold injuries in sleep‐deprived subjects. This finding needs to be confirmed by epidemiological studies.

During the CWI, we did not observe a significant effect of TSD on the mean temperatures of fingers 2 and 4 in the placebo or caffeine condition, as described in our previous study after TSD (Sauvet et al., 2012). However, the significant interaction between the day and treatment effects on minimal finger temperatures indicates that this temperature decreases after TSD in the placebo condition. The CWI duration and the cooled surface area might explain the lack of effect of TSD on mean finger temperature, but we voluntarily limited the duration of immersion to 20 min because we wanted to limit the duration of pain, especially for subjects having no CIVD. We also chose to immerse only the fingers because of their more pronounced CIVD compared with the hand or forearm (Sendowski et al., 1997) and because this would be less painful and limit the overall effects of CWI on BCT, arm temperature and the stress response (Daanen, 2003). A recent numerical study evidenced that the convective heat transfer coefficients of the five fingers are larger than those of the palm, dorsal hand and wrist, and the fingertip is the section most vulnerable to heat loss when the hand is exposed to a cold environment (Zhang, 2021; Zhang et al., 2021).

During the rewarming period after CWI, high perfusion values (indicated by SkBF) are observed very quickly after removal of the fingers from the cold water, drying them and placing them under the speckle laser (i.e., after 1 min), decreasing after 5 min. This response profile for variance (disparity) of SkBF changes between fingers is broadly identical. Furthermore, the rewarming responses are sensitive to TSD, with lower perfusion values after 1 min in both placebo and caffeine conditions and with higher variance over virtually all measurement time points. This increase of the disparity between fingers has been described previously for temperatures in patients with Raynaud's phenomenon, as a useful thermographic indicator for evaluating disturbed peripheral circulation (Horikoshi et al., 2016). Our results illustrate the pertinence of the use of laser speckle contrast imaging for the monitoring of blood perfusion during the rewarming period after CWI. In the dynamic assessment of digital vascular perfusion, laser speckle values correlate well with those of infrared thermography (i.e., skin surface temperatures) in healthy subjects (Pauling et al., 2012) and were shown to be reproducible and sensitive to temperature reduction in a cold challenge of the hand in patients with sclerosis‐related Raynaud's phenomenon (Wilkinson et al., 2018).

The reduced response to cold after TSD could be linked to mechanisms including vascular (hypothesized vasoconstrictor and vasodilator alterations), hormonal or immuno‐inflammatory responses triggered by TSD, as demonstrated in several previous studies (Pagani et al., 2009; Sauvet et al., 2010). We have consolidated the TSD‐induced endothelial dysfunction, occurring well before the changes in systolic blood pressure, HR and sympathetic activity in human (Sauvet et al., 2014). Endothelial function could be a key factor in the mechanisms of CIVD and rewarming after cold immersion (Daanen, 2003); however, the CIVD response was recently found to be related to neural activity (i.e., a consequence of sympathetic vasoconstrictor withdrawal; Flouris & Cheung, 2009; Hodges et al., 2018), but not endothelium‐mediated mechanisms (no change of endothelial nitric oxide‐dependent and ‐independent activities; Hodges et al., 2018). For Hodges et al. (2018), the neural activity seems consistently to be reduced prior to CIVD, suggesting that sympathetic withdrawal directly contributes to CIVD onset. The link between CIVD and endothelial function remains to be clarified.

In this study, we evidenced that the acute caffeine intake increases fingers temperatures (the mean and minimal) during CWI and rewarming (the mean and maximal) after TSD particularly, and the effect is also present on the mean temperature during CWI before TSD. The increase in the finger temperatures by caffeine after TSD during the rewarming period is concomitant to a decrease in SkBF disparity at the 5, 10 and 15 min points. The effects of caffeine after TSD are, however, more contrasted on perfusion values with time‐limited increases (1 and 5 min). The protective effect of acute caffeine on the mean finger temperatures during the CWI period before TSD contrasts with a previous study showing that caffeine intake resulted in a lower mean finger temperature during CWI in a chair‐rest condition (i.e., not exercising) in healthy non‐regular caffeine consumers (Kim et al., 2013). However, in their study: (1) the amount of caffeine administered in the 30 min before CWI (300 mg per dose) was larger than in our study [∼175 mg for 70 kg (2.5 mg/kg per dose), administered 90 min before CWI] and provided by a chewing gum, offering a different pharmacokinetic profile from the ingestion of caffeine powder in a decaffeinated beverage, as in our study; and (2) the significant effect of caffeine on CIVD was evidenced after resting, whereas it was also after TSD in our study, and our subjects were regular but moderate caffeine consumers. This protective effect of caffeine was also observed on the mean and maximal temperatures in the rewarming period after TSD.

In our study, there was a significant day effect on pain during the CWI (from the onset to the end) and at the 2 min rewarming time, with a significant interaction with treatment (during CIVD, end of CWI and rewarming at 2 and 20 min). The pain was significantly increased during CWI and rewarming after TSD in the caffeine condition, but not in the placebo condition. At the same points, except for the 20 min rewarming, pain was significantly decreased after caffeine intake before TSD. This result suggests a beneficial effect of caffeine on pain as long as there is no constraint of TSD, with a possible induction of perceived pain after TSD under the effect of caffeine intake. The increased of pain sensitivity to cold pain stimuli has been described after TSD (Staffe et al., 2019). The acute beneficial effect of caffeine has been described on pain tolerance and pain thresholds in normotensive men and women in a cold pressor test (hand in an ice water bath, maintained at a temperature between 0°C and 3°C) 45 min after acute caffeine intake (250 mg) (Keogh & Witt, 2001). To our knowledge, there are no studies describing a beneficial effect of caffeine on pain tolerance during a CWI test in healthy subjects. This analgesic effect of caffeine before TSD could be a consequence of its effect of increasing the mean finger temperature during CWI.

Caffeine is a xanthine with several peripheral and central effects that can act on vascular tissue. It acts directly on the endothelial cell, stimulating the production of NO by increasing intracellular calcium, and the NO diffuses into the vascular smooth muscle cell to produce vasodilatation (Cappelletti et al., 2015; Echeverri et al., 2010). The vasodilatation in vascular smooth muscle cells implicates predominantly a competitive inhibition of phosphodiesterase, producing an accumulation of cAMP. Another mechanism of caffeine action is the antagonism of adenosine receptors present in the vascular tissue, which can lead to vasoconstriction (Echeverri et al., 2010). However, the relationship between caffeine and endothelial function is controversial. Umemura et al. (2006) found that caffeine (300 mg) significantly increased both systolic and diastolic blood pressures but did not alter HR and baseline forearm blood flow in healthy young men. In their study, caffeine augmented the ACh‐induced (endothelium‐dependent) vasodilatation, whereas sodium nitroprusside‐induced (endothelium‐independent) vasodilatation was not altered. In our study, the total daily caffeine intake (2 × 175 mg in the day, at 08.30 and 14.30 h, i.e., a moderate dose) increased finger skin temperature before TSD during the CWI test performed at 16.00 h probably increased SkBF (as suggested at 1 and 5 min of rewarming), without effects on cardiovascular parameters. We suggest that the effect of caffeine to increase finger temperatures and perfusion before TSD might have facilitated their increase in rewarming after TSD. Thus, it is not unreasonable to speculate that the protective effect of acute caffeine on the local cold response, particularly in regular but moderate (∼250 mg/day) caffeine consumers, could be linked to a vasodilatory effect of caffeine, and not to an immediate vasoconstrictive effect of caffeine acting as an adenosine receptor antagonist (Fredholm & Persson, 1982; Hom & Lokhandwala, 1981). Taken together, our results suggest that moderate (350 mg in the day before the CWI test) caffeine intake could have a beneficial effect on cold tolerance through a potential acute stimulation of vasodilatory mechanisms. However, the significant negative correlation between habitual daily caffeine consumption and minimal temperatures before and after TSD in our study suggests that the limit of the protective effect of acute caffeine intake would depend on daily consumption. This might be linked to the impact of chronic caffeine consumption on the adenosine receptor systems. Several studies on receptor binding have shown either upregulation of A_1_ or A_2A_ receptors or an increase in the affinity of either system, suggesting differences with acute versus chronic treatment (Jacobson et al., 1996).

This study had several limitations, both in the conditions of the CWI test (in terms of duration, in particular) and in the acute administration of caffeine (especially in terms of the dose chosen). These limits restrict the advice that could be provided regarding the tolerance of the fingers/hand to cold and the consumption (both occasional and chronic) of caffeine. First, we used moderate caffeine administration, corresponding to the average estimate of habitual daily caffeine consumption in a middle‐aged French working population (i.e., 225 ± 165 mg, *n* = 1498; Sanchez‐Ortuno et al., 2005). We excluded subjects usually consuming >500 mg/day of caffeine in order to prevent adverse effects induced by withdrawal in the placebo condition (headache and cognitive disorders; Hughes et al., 1991) and/or possible cardiovascular effects (in addition to those related to the stress of the CWI test and/or TSD). Indeed, using >500 mg caffeine is associated with side‐effects and high levels of sleep disorders (Hughes et al., 1991; Johnson‐Greene et al., 1988). The second limitation of this study is the relatively low number of subjects permitting the analysis of the incidence (interaction) of the habitual daily caffeine consumption on the acute effects of caffeine intake. Nevertheless, this was not the primary aim of this work. A third limitation is that we did not study cold‐induced injuries but tolerance to cold in a laboratory environment. Further ecological studies are needed to confirm our results. Recent data from a military arctic training exercise showed the limits of recorded values during CWI as predictors of cold injury during ecological or operational environments (Sullivan‐Kwantes et al., 2019).

## 5 CONCLUSION

In this controlled laboratory study on 36 healthy subjects, we described the effects of acute caffeine on local (fingers) cold tolerance after TSD through measurements of skin temperatures, skin perfusion and perceived pain using the CWI test (20 min, 5°C) followed by a 20 min rewarming period to ambient air. We partly confirmed our previous study on the decreased cold tolerance after TSD (Sauvet et al., 2012). We also demonstrated, in a double‐blind study, that moderate caffeine intake (vs. placebo) (2.5 mg/kg twice daily) improves cold tolerance and passive rewarming after and before TSD. In contrast, high (i.e., daily) caffeine consumers had lower minimal finger temperatures during CWI. Further studies are needed to confirm the potential pathophysiological effects of high habitual daily caffeine consumption. Taken together, our results provide opportunities for new recommendations related to acute and chronic caffeine consumption, particularly in a cold environment.

## AUTHOR CONTRIBUTIONS

Baptiste de Lorgeril, Pierre‐Emmanuel Tardo‐Dino, Michael Quiquempoix, Lise Mateo, Philippe Colin and Pascal Van Beers: data acquisition, analysis, interpretation, drafting and revising of the manuscript. Fabien Sauvet, Mégane Erblang, Cyprien Bourrilhon, Catherine Drogou, Danielle Gomez‐Merino and Mounir Chennaoui: design, interpretation of data, drafting and revising the manuscript critically for important intellectual content. All authors approved the final version of the manuscript and agree to be accountable for all aspects of the work in ensuring that questions related to the accuracy or integrity of any part of the work are appropriately investigated and resolved. All persons designated as authors qualify for authorship, and all those who qualify for authorship are listed.

## CONFLICT OF INTEREST

None declared.

## Data Availability

Data could be obtained by asking the corresponding author.

## Impact of acute caffeine intake on local tolerance to cold before and after total sleep deprivation

Baptiste de Lorgeril, Pierre‐Emmanuel Tardo‐Dino, Cyprien Bourrilhon, Michael Quiquempoix, Catherine Drogou, Lise Mateo, Mégane Erblang, Philippe Colin, Pascal Van Beers, Mounir Chennaoui, Danielle Gomez‐Merino, Fabien Sauvet.

**TABLE A1:** Average values of body core temperature, thenar and hypothenar eminence temperatures, systolic, diastolic and mean arterial pressures and heart rate, during pre‐immersion, 5°C cold‐water immersion and rewarming.

		Pre‐immersion		CWI		Rewarming	
Parameter	Condition	Before TSD	After TSD	Before TSD	After TSD	Before TSD	After TSD
Body core temperature (°C)	Placebo	37.30 ± 0.61	37.04 ± 0.32	37.08 ± 0.31 (*p* = 0.98)	37.25 ± 0.42 (*p* = 0.89)	37.12 ± 0.51 (*p* = 0.87)	37.54 ± 0.38 (*p* = 0.78)
	Caffeine	37.25 ± 0.48	37.21 ± 0.25	37.24 ± 0.38 (*p* = 0.89)	37.29 ± 0.29 (*p* = 0.82)	37.32 ± 0.56 (*p* = 0.87)	37.55 ± 0.25 (*p* = 0.76)
E Th (°C)	Placebo	29.98 ± 2.48	30.29 ± 2.85	23.67 ± 3.01 (*p* = 0.02)	23.98 ± 3.25 (*p* = 0.02)	22.46 ± 3.38 (*p* = 0.01)	23.69 ± 3.43 (*p* = 0.02)
	Caffeine	30.62 ± 2.57	30.97 ± 1.99	26.21 ± 3.03 (*p* = 0.02)	24.27 ± 3.15 (*p* = 0.02)	23.58 ± 5.58 (*p* = 0.01)	23.77 ± 5.89 (*p* = 0.01)
E HTh (°C)	Placebo	30.35 ± 2.36	30.45 ± 2.36	23.99 ± 2.86 (*p* = 0.01)	24.06 ± 2.28 (*p* = 0.02)	22.52 ± 2.91 (*p* = 0.01)	23.92 ± 4.40 (*p* = 0.01)
	Caffeine	30.91 ± 2.49	30.69 ± 2.70	26.48 ± 3.94 (*p* = 0.02)	24.23 ± 2.84 (*p* = 0.02)	24.06 ± 2.62 (*p* = 0.02)	23.47 ± 2.63 (*p* = 0.02)
SAP (mmHg)	Placebo	116.18 ± 18.96	117.49 ± 12.05	134.88 ± 15.45 (*p* = 0.002)	136.04 ± 15.46 (*p* = 0.004)	119.08 ± 12.34 (*p* = 0.12)	119.63 ± 18.38 (*p* = 0.21)
	Caffeine	125.23 ± 12.13	123.01 ± 12.13	139.85 ± 15.46 (*p* = 0.004)	142.03 ± 15.60 (*p* = 0.003)	124.50 ± 12.45 (*p* = 0.62)	121.76 ± 18.45 (*p* = 0.62)
DAP (mmHg)	Placebo	68.27 ± 6.44	70.79 ± 6.47	82.85 ± 12.36 (*p* = 0.02)	83.34 ± 12.31 (*p* = 0.02)	72.17 ± 6.83 (*p* = 0.12)	73 93 ± 6.87 (*p* = 0.23)
	Caffeine	68.73 ± 6.60	71.47 ± 6.51	80.71 ± 12.36 (*p* = 0.02)	83.19 ± 12.34 (*p* = 0.02)	73.26 ± 6.87 (*p* = 0.34)	76.23 ± 6.91 (*p* = 0.24)
MAP (mmHg)	Placebo	84.24 ± 18.78	86.36 ± 18.86	94.22 ± 15.29 (*p* = 0.03)	100.91 ± 15.39 (*p* = 0.03)	87.81 ± 18.96 (*p* = 0.12)	88.73 ± 18.95 (*p* = 0.18)
	Caffeine	80.82 ± 18.78	81.83 ± 18.78	91.93 ± 15.30 (*p* = 0.03)	94.90 ± 15.23 (*p* = 0.03)	83.79 ± 18.93 (*p* = 0.22)	84.27 ± 18.99 (*p* = 0.21)
Heart rate (beats/min)	Placebo	77.81 ± 19.26	78.75 ± 24.04	91.23 ± 12.43 (*p* = 0.02)	90.40 ± 12.48 (*p* = 0.02)	75.22 ± 12.39 (*p* = 0.43)	75.28 ± 12.44 (*p* = 0.32)
	Caffeine	73.97 ± 19.96	74.38 ± 19.65	89.25 ± 12.53 (*p* = 0.01)	91.25 ± 12.53 (*p* = 0.02)	77.50 ± 12.47 (*p* = 0.23)	75.58 ± 12.49 (*p* = 0.22)

*Note*: Values are means ± SD. The *p*‐values are expressed in comparison to pre‐immersion values.

Abbreviations: DAP, diastolic arterial pressure; E Th and E HTh, thenar and hypothenar eminence temperatures; MAP, mean arterial pressure; SAP, systolic arterial pressure; TSD, total sleep deprivation.

**TABLE A2:** Statistical results of the mixed model during pre‐immersion.

		BCT		E Th		E HTh		SAP		MAP		DAP		HR	
Effects	d.f.	*F*	*p*‐value	*F*	*p*‐value	*F*	*p*‐value	*F*	*p*‐value	*F*	*p*‐value	*F*	*p*‐value	*F*	*p*‐value
Day	1	1.16	0.44	0.93	0.33	0.01	0.92	0.45	0.50	0.83	0.36	1.81	0.18	0.08	0.78
Treatment	1	1.01	0.32	3.78	0.06	2.72	0.12	2.08	0.15	1.79	0.18	0.11	0.75	2.39	0.12
Caffeine consumption	2	0.56	0.36	0.92	0.26	0.15	0.70	3.33	0.07	2.60	0.11	2.57	0.11	0.23	0.64
Day × Treatment	1	1.23	0.39	0.01	0.98	0.49	0.49	1.08	0.30	1.01	0.32	0.15	0.70	0.08	0.78
Day × Caffeine consumption	2	0.70	0.25	0.67	0.41	0.72	0.40	0.87	0.35	0.65	0.42	1.18	0.27	0.37	0.54
Treatment × Caffeine consumption	2	0.67	0.41	0.67	0.41	0.14	0.69	0.97	0.32	1.61	0.21	0.08	0.78	2.90	0.09
Day × Treatment × Caffeine consumption	2	0.21	0.24	1.40	0.29	0.16	0.69	0.99	0.33	0.39	0.54	0.20	0.66	0.01	0.91

*Note*: ‘Day’ is after total sleep deprivation versus before. ‘Treatment’ is caffeine condition versus placebo. ‘Caffeine consumption’ is the habitual daily caffeine consumption. Abbreviations: BCT, body core temperature; DAP, diastolic arterial pressure; E Th and E HTh, thenar and hypothenar eminence temperatures; HR, heart rate; MAP, mean arterial pressure; SAP, systolic arterial pressure.

**TABLE A3:** Statistical results of the mixed model during 5°C cold‐water immersion.

		BCT		E Th		E HTh		SAP		MAP		DAP		HR	
Effects	d.f.	*F*	*p*‐value	*F*	*p*‐value	*F*	*p*‐value	*F*	*p*‐value	*F*	*p*‐value	*F*	*p*‐value	*F*	*p*‐value
Day	1	0.19	0.66	0.65	0.42	1.74	0.18	1.74	0.19	3.70	0.16	0.01	0.92	0.93	0.33
Treatment	1	0.65	0.42	0.44	0.51	0.32	0.57	0.32	0.57	0.04	0.84	0.72	0.40	3.78	0.05
Caffeine consumption	2	0.36	0.55	0.93	0.34	1.49	0.22	1.64	0.15	3.09	0.08	1.26	0.22	1.13	0.28
Day × Treatment	1	0.09	0.76	0.06	0.81	10.7	0.39	1.49	0.22	0.03	0.86	0.15	0.70	0.01	0.91
Day × Caffeine consumption	2	0.37	0.55	1.01	0.32	1.85	0.17	1.07	0.30	0.18	0.67	0.49	0.49	0.01	0.92
Treatment × Caffeine consumption	2	2.03	0.16	0.01	0.91	0.64	0.42	1.85	0.18	4.38	0.14	0.72	0.40	0.67	0.41
Day × Treatment × Caffeine consumption	2	0.01	0.93	0.01	0.91	0.61	9.43	0.64	0.42	0.01	0.91	0.16	0.69	0.01	0.96

*Note*: ‘Day’ is after total sleep deprivation versus before. ‘Treatment’ is caffeine condition versus placebo. ‘Caffeine consumption’ is the habitual daily caffeine consumption. Abbreviations: BCT, body core temperature; DAP, diastolic arterial pressure; E Th and E HTh, thenar and hypothenar eminence temperatures; HR, heart rate; MAP, mean arterial pressure; SAP, systolic arterial pressure.

**TABLE A4:** Statistical results of the mixed model during rewarming after cold‐water immersion.

		BCT		E Th		E HTh		SAP		MAP		DAP		HR	
Effects	d.f.	*F*	*p*‐value	*F*	*p*‐value	*F*	*p*‐value	*F*	*p*‐value	*F*	*p*‐value	*F*	*p*‐value	*F*	*p*‐value
Day	1	1.58	0.21	0.45	0.50	0.83	0.36	0.08	0.78	1.81	0.19	0.71	0.40	1.58	0.21
Treatment	1	0.01	0.99	2.08	0.15	1.79	0.18	0.39	0.53	0.11	0.74	0.37	0.55	0.02	0.99
Caffeine consumption	2	0.65	0.42	3.33	0.07	2.60	0.11	0.23	0.63	2.57	0.11	0.75	0.39	0.65	0.42
Day × Treatment	1	0.05	0.83	1.08	0.30	1.01	0.32	0.08	0.78	0.25	0.70	0.37	0.55	0.05	0.83
Day × Caffeine consumption	2	0.29	0.59	0.87	0.35	0.65	0.42	0.37	0.54	1.18	0.27	0.12	0.73	0.29	0.58
Treatment × Caffeine consumption	2	1.71	0.41	0.97	0.33	1.61	0.21	2.90	0.09	0.08	0.78	1.20	0.28	1.71	0.41
Day × Treatment × Caffeine consumption	2	0.02	0.95	0.99	0.33	0.39	0.54	0.01	0.91	0.20	0.65	0.10	0.75	0.02	0.95

*Note*: ‘Day’ is after total sleep deprivation versus before. ‘Treatment’ is caffeine condition versus placebo. ‘Caffeine consumption’ is the habitual daily caffeine consumption. Abbreviations: BCT, body core temperature; DAP, diastolic arterial pressure; E Th and E HTh, thenar and hypothenar eminence temperatures; HR, heart rate; MAP, mean arterial pressure; SAP, systolic arterial pressure.
